# Retosiban Prevents Stretch-Induced Human Myometrial Contractility and Delays Labor in Cynomolgus Monkeys

**DOI:** 10.1210/jc.2017-02195

**Published:** 2017-12-26

**Authors:** Irving L M H Aye, Alexandros A Moraitis, Dinesh Stanislaus, D Stephen Charnock-Jones, Gordon C S Smith

**Affiliations:** 1 Department of Obstetrics and Gynaecology, University of Cambridge, National Institute for Health Research, Cambridge Comprehensive Biomedical Research Centre, Cambridge, United Kingdom; 2 Department of Reproductive Toxicology, GlaxoSmithKline, Philadelphia Navy Yard, Philadelphia, Pennsylvania

## Abstract

**Context:**

Stretch of the myometrium promotes its contractility and is believed to contribute to the control of parturition at term and to the increased risk of preterm birth in multiple pregnancies.

**Objective:**

To determine the effects of the putative oxytocin receptor (OTR) inverse agonist retosiban on (1) the contractility of human myometrial explants and (2) labor in nonhuman primates.

**Design:**

Human myometrial biopsies were obtained at planned term cesarean, and explants were exposed to stretch in the presence and absence of a range of drugs, including retosiban. The *in vivo* effects of retosiban were determined in cynomolgus monkeys.

**Results:**

Prolonged mechanical stretch promoted myometrial extracellular signal-regulated kinase (ERK)1/2 phosphorylation. Moreover, stretch-induced stimulation of myometrial contractility was prevented by ERK1/2 inhibitors. Retosiban (10 nM) prevented stretch-induced stimulation of myometrial contractility and phosphorylation of ERK1/2. Moreover, the inhibitory effect of retosiban on stretch-induced ERK1/2 phosphorylation was prevented by coincubation with a 100-fold excess of a peptide OTR antagonist, atosiban. Compared with vehicle-treated cynomolgus monkeys, treatment with oral retosiban (100 to 150 days of gestational age) reduced the risk of spontaneous delivery (hazard ratio = 0.07, 95% confidence interval 0.01 to 0.60, *P* = 0.015).

**Conclusions:**

The OTR acts as a uterine mechanosensor, whereby stretch increases myometrial contractility through agonist-free activation of the OTR. Retosiban prevents this through inverse agonism of the OTR and, *in vivo*, reduced the likelihood of spontaneous labor in nonhuman primates. We hypothesize that retosiban may be an effective preventative treatment of preterm birth in high-risk multiple pregnancies, an area of unmet clinical need.

## Teaser

We studied the effects of retosiban, a putative OTR inverse agonist in human myometrial explants and cynomolgus monkeys. We found that the OTR acts as a uterine mechanosensor, blocked by retosiban.

Multiple gestations (twins and higher order births) comprise 3% to 4% of all births in the United States (1) but contribute disproportionately to preterm birth and its related complications. Infant mortality is 4, 12, and 25 times higher in twins, triplets, and quadruplets, respectively (2), and these associations are largely due to an increased risk of spontaneous preterm birth. Costs of pediatric care in the United States (2005 to 2010) were $66,000 more following twin birth (compared with singleton) and $336,000 more for higher multiples (2).

There is a large body of evidence to indicate that increasing mechanical stretch of the myometrium promotes its contractility. As well as contributing to the physiological initiation of parturition at term (3), uterine stretch is also thought to account for the association between multiple pregnancy and spontaneous preterm birth (4). Consistent with this hypothesis, other clinical conditions associated with increased mechanical stretch of the uterine wall in singleton pregnancies, such as polyhydramnios, are also associated with an increased risk of preterm labor (5, 6). However, there is currently no clearly effective therapeutic approach to women with multiple pregnancy who are at high risk of spontaneous preterm birth (7).

The oxytocin receptor (OTR) system plays an important role in the regulation of myometrial contractility. OTR levels are tightly regulated during pregnancy, peaking upon labor onset, followed by a sharp decline in the postpartum period when the uterus becomes refractory to oxytocin (8). Furthermore, mechanical stretch of the uterus has been proposed as a major physiological stimulus regulating the OTR system during pregnancy (9, 10). We previously demonstrated that the stimulatory effects of mechanical stretch on contractility of human myometrial explants were inhibited by 1 µM retosiban (GSK221149A) (11), a nonpeptide, orally active, and selective OTR antagonist (12). In the current study, we test the hypotheses that stretch increases myometrial contractility through agonist-free activation of the OTR and that this is inhibited by retosiban through inverse agonism of the OTR. We also sought to determine whether the drug alters the timing of spontaneous labor when administered orally to a nonhuman primate.

## Materials and Methods

### Study subjects and tissue collection

Human myometrial samples were obtained from a total of 85 nonlaboring women with normal term pregnancies, undergoing routine elective cesarean section. All patients gave their informed, written consent to participate, and the study was approved by the Cambridge Research Ethics Committee (04/Q0108/290). The specimens were taken from the upper edge of the lower uterine segment incision following delivery of the baby and the placenta. Myometrial samples were placed in Krebs solution on ice and processed within 10 minutes of delivery.

### Myometrial explant culture and experimental design

Each myometrial biopsy was cleared of the serosa, fibrous tissue, and blood vessels, and dissected into eight longitudinal explants of ∼2 to 3 × 8 to 12 mm. The explants were maintained in culture medium composed of phenol red–free Dulbecco modified Eagle medium supplemented with 10% charcoal-stripped fetal bovine serum, 2 mM l-glutamine, and antibiotic/antimycotic solution (Sigma-Aldrich, St. Louis, MO). The explants were suspended in culture medium under either low tension (0.6 g mass) or high tension (2.4 g mass) using the method previously described (11, 13). The explants were then treated with retosiban (0.1 nM to 1 µM; GlaxoSmithKline, Stevenage, United Kingdom), atosiban (10 nM to 1 µM; Bowmed Ibisqus Ltd., Wrenham, United Kingdom), U0126 (10 µM; Tocris Bioscience, Abingdon, United Kingdom), SCH772984 (10 µM; Tocris Bioscience), or vehicle (dimethyl sulfoxide, 0.1% v/v), and incubated in a 5% CO_2_ humidified atmosphere at 37°C. Following 20-hour incubation, the explants were transferred to an organ bath for isometric tension studies or processed for protein analyses. For inverse agonism studies, explants were incubated with either 10 nM retosiban, 1 µM atosiban, or retosiban with atosiban. Retosiban exhibits ∼16-fold greater affinity for the OTR than atosiban (14); hence, a 100-fold excess of retosiban was used to determine whether the atosiban could block the effects of 10 nM retosiban.

### Isometric tension measurements

Myometrial contractility was studied using the previously described protocol (15), and all explants were studied under 2 g tension. For analysis of contractility after explant culture, maximal responses to KCl and oxytocin (measured in grams) were normalized to strip wet weight (also measured in grams) to produce a normalized response. The mean normalized responses of duplicate strips were then calculated. Effects were expressed as fold change, *i.e.*, the ratio of the mean normalized responses in the experimental and control conditions from different strips obtained from the same woman. Negative logarithm of the half-maximal effective concentration (pEC_50_) values were calculated using analysis of the area under the curve for each concentration to oxytocin, as previously described (16). The concentration-dependent effects of retosiban were studied in myometrial biopsies from 20 different women, and the effects of mitogen-activated protein kinase kinase (MEK)/extracellular signal-regulated kinase (ERK) inhibitors were studied in myometrial biopsies from 10 different women.

### Phosphokinase array analysis

Analysis of the phosphorylation profiles of kinases and their protein substrates was performed using Human Phospho-Kinase Arrays (R&D Systems, Minneapolis, MN). Myometrial explants (N = 12) were incubated for 20 hours under 0.6 g or 2.4 g with vehicle for 20 hours and then snap frozen in liquid nitrogen and transferred to −80°C. Protein lysates were prepared using the FastPrep24 sample disruption system with Lysing Matrix S tubes (MP Biomedicals, Santa Ana, CA). Protein concentration was determined by bicinchoninic acid assay (ThermoFisher Scientific, Waltham, MA), and 500 μg each tissue lysate was added to preblocked antibody array membranes for incubation. Membranes were treated with detection antibody cocktail, followed by streptavidin-horseradish peroxidase as instructed, and signal was detected using enhanced chemiluminescence (ECL) on Kodak X-Omat LS X-ray film (Sigma-Aldrich). ECL detection was performed at multiple time points to acquire the optimal exposure period for different antibody targets. Signal intensities were quantified using ImageJ software (imagej.nih.gov) and calculated as instructed in the array manufacturer’s protocol.

### Protein phosphorylation and oxytocin analysis by enzyme-linked immunosorbent assays

Enzyme-linked immunosorbent assays (ELISAs) were performed to validate the results obtained from phosphokinase arrays, in an independent set of myometrial explants subjected to either 0.6 g or 2.4 g tension for 20 hours and incubated with retosiban (0.1 nM to 1 µM), atosiban (10 nM to 1 µM), and 10 nM retosiban with 1 µM atosiban or vehicle (N = 33). The following ELISAs were performed according to the manufacturer’s instructions: DuoSet IC ELISAs (R&D Systems), phospho-ERK1 (T202/Y204)/ERK2 (T185/Y187), total *β*-catenin and phospho–platelet-derived growth factor receptor *β* (PDGF Rb), and Simple Step ELISA (Abcam, Cambridge, United Kingdom) phospho-STAT5A/B (Y694/699) and phospho-STAT5A (Y694). Oxytocin ELISAs were performed in lysates of myometrial explants subjected to 0.6 g or 2.4 g tension. Source of the ELISA kits and their catalog numbers are listed in Supplemental Table 1.

### Western blot analysis

ERK phosphorylation data from tissue ELISAs were confirmed by Western blot analysis using the same phospho-ERK1 (T202/Y204) ERK2 (T185/Y187) antibody clone (R&D Systems). Myometrial explant cultures (20 hours) were performed with the following treatments: 0.6 g vehicle, 2.4 g vehicle, and 2.4 g with 10 nM retosiban. Frozen tissues were lysed in radioimmunoprecipitation assay buffer containing protease inhibitors and phosphatase inhibitor cocktail 1 and 2 (1:100; Sigma-Aldrich). Protein concentrations were determined by bicinchoninic acid assay (ThermoFisher Scientific). Twenty micrograms of protein was loaded into each well and separated on Any KD Mini-Protean Tris Glycine Precast gels (Bio-Rad, Hercules, CA). Separated proteins were then transferred onto polyvinylidene difluoride membranes using the iBlot system (ThermoFisher Scientific) and blocked with 5% nonfat milk powder for 1 hour. After washing in Tris-buffered saline containing 0.1% Tween (TBS-T), membranes were incubated in rabbit anti–phospho-ERK1 (T202/Y204) ERK2 (T185/Y187) antibody (R&D Systems) at 1 µg/mL overnight at 4°C in TBS-T containing 2% bovine serum albumin. The membranes were then washed and incubated with peroxidase-conjugated goat anti-rabbit (1:2000) in TBS-T with 2% bovine serum albumin for 2 hours at room temperature and detected by ECL using ECL Western Blotting Substrate (Pierce, ThermoFisher Scientific) on Kodak X-Omat LS X-ray film (Sigma-Aldrich). Membranes were stripped in Restore Western Blot Stripping Buffer (ThermoFisher Scientific) and reprobed with mouse anti-ERK1/2 antibody (Cell Signaling Technology, Danvers, MA). Densitometry analysis was performed using ImageJ software. Total protein levels were determined by Amido Black (Sigma-Aldrich) staining.

### Toxicological experiments on cynomolgus monkeys

All animal studies were ethically reviewed and carried out in accordance with European Directive 2010/63/EEC and the GlaxoSmithKline Policy on the Care, Welfare, and Treatment of Animals. Sufficient purpose-bred cynomolgus monkeys (*Macaca fascicularis*) of Chinese/Vietnamese origin were obtained from Nafo Vanny (Vietnam) and Guangdong Scientific Instruments (China). The female animals were purpose bred and sexually mature. The females were in the weight range from 2.5 to 6.6 kg at selection for mating and were at least 3 years old. Sexually mature, untreated males were used for mating only. Animals were housed in individual cages, and the environmental controls were set to maintain temperature within the range 19°C to 25°C and relative humidity within the range 30% to 70% with a ∼12-hour light and ∼12-hour dark cycle. Each animal was offered twice daily a commercial pellet diet for primates (Ssniff P10, Ssniff Spezialdiäten, Ferdinand-Gabriel-Weg 16, 59494 Soest, Germany). In addition, the animals received fresh fruit and bread. Any residual food was removed and estimated in the morning and afternoon. Tap water was provided *ad libitum* via an automatic watering system or bottles.

The data presented in this work are part of a toxicology study conducted to assess the safety of retosiban, and not all data and end points are described. The animals were assigned to dosing groups on day 90 *postcoitum* using a random table. By use of this random table, the sequence of pregnant animals assigned to treatment groups was predetermined. Pregnant animals were selected to the study based on day 90 *postcoitum* ultrasound to confirm the pregnancy, absence of any signs of ill health of the mother or the fetus, within the normal range gestational body weight gain, and fetal size within normal range. Retosiban or vehicle [1% (w/v) aqueous methylcellulose with 0.1% (w/v) Tween 80] was given by oral administration (4 mL/kg/d) once per day to the pregnant animals between day 100 and 150 of gestation (0.6 and 0.9 gestation) at either 100 mg/kg/d or 300 mg/kg/d. The average duration of cynomolgus gestation in a primate facility is 160 days (17).

Each of the three groups (vehicle, 100 mg/kg/d, or 300 mg/kg/d) included 18 animals: 12 were allowed to progress to labor, and 6 were delivered by planned cesarean at 150 ± 1 days. One of the purposes of planned cesarean section was to allow collection of fetal blood from the umbilical vein to determine the concentration of retosiban. Maternal blood was also collected from the anesthetized mother immediately after fetal blood collection to determine maternal retosiban concentrations. Of the 18 animals (6 from each group) in which prelabor cesarean delivery was planned, 2 vehicle control animals delivered spontaneously and a single retosiban animal (300 mg/kg/d retosiban) had an emergency cesarean section performed on gestation day 148 due to signs associated with delivery (hunched posture). In the statistical analysis, this delivery was treated as spontaneous labor rather than censoring. The remaining 36 monkeys were allowed to deliver their offspring, and gestation length was calculated. Hence, overall, 38 animals had a spontaneous delivery, one had an emergency cesarean delivery for presumed labor, and 15 animals had a planned prelabor cesarean.

Although we do not present any safety data in the present paper, a range of endpoints related to the safety of retosiban for primate pregnancy and offspring health was evaluated. In pregnant monkeys, clinical signs, ultrasonography, fetal heart rates, weekly body weight, and food consumption were assessed. At cesarean delivery, the fetal blood pH, hematocrit, hemoglobin, blood gas, placental weight and morphology, fetal weight, external morphology and gross organ morphology, and microscopic evaluation of kidney and lung were assessed. For the spontaneously delivered animals, the mother and the offspring were evaluated for 6 months, and during this time offspring electrocardiogram and neuromuscular development were assessed at 5 to 6 weeks of age along with body weights and clinical observations. Infant blood sampling (blood gasses, pH, clinical pathology) was conducted at 3 months of age. There were no retosiban-related effects on any of the experimental endpoints evaluated that precluded the administration of retosiban to pregnant women. The safety data were reviewed by both the US Food and Drug Administration and the European Union European Medicines Agency prior to approval for human clinical trials of retosiban (NCT02377466, NCT02292771, and NCT00404768) in the treatment of women presenting in presumed spontaneous preterm labor, the original indication for the drug.

### Statistical analysis

For human myometrial studies, all experiments were repeated in myometrial biopsies from 10 to 20 different women (represented by N). The data are expressed as fold changes, ratios were log transformed, and the normality of the distribution following log transformation was assessed using Shapiro–Wilk test. For contractility and ELISA experiments, paired *t* test or one-way ANOVA followed by Dunnett *post hoc* test was performed, and *P* < 0.05 was considered significant. Phosphokinase array experiments were analyzed by multiple *t* tests and corrected for false discovery rate using the Benjamini–Hochberg method (false discovery rate set at 5%).

The primate data were analyzed using time-to-event methods, as previously described (18). The event was spontaneous delivery (or, in a single case, emergency cesarean during labor), and planned cesarean was treated as censored. Data were presented using a Kaplan–Meier plot. Modeling was by the Cox proportional hazards model, and the proportional hazards assumption was tested using the method of Grambsch and Therneau (19). Given that visual inspection of the Kaplan–Meier plot indicated crossing of the curves and that the proportional hazards test was significant, the comparison of the Kaplan–Meier plot used the Wilcoxon (Breslow) test (20), as the more conventionally used log–rank test tends to be underpowered in this situation (21). To determine whether the results were dependent on the specific test used, we also compared the curves using two other tests that are appropriate when the hazards are nonproportional: the Tarone-Ware and Peto-Peto-Prentice tests (22, 23).

## Results

### Prolonged mechanical stretch stimulates myometrial contractility and activates phosphokinase proteins

**Figure 1.**
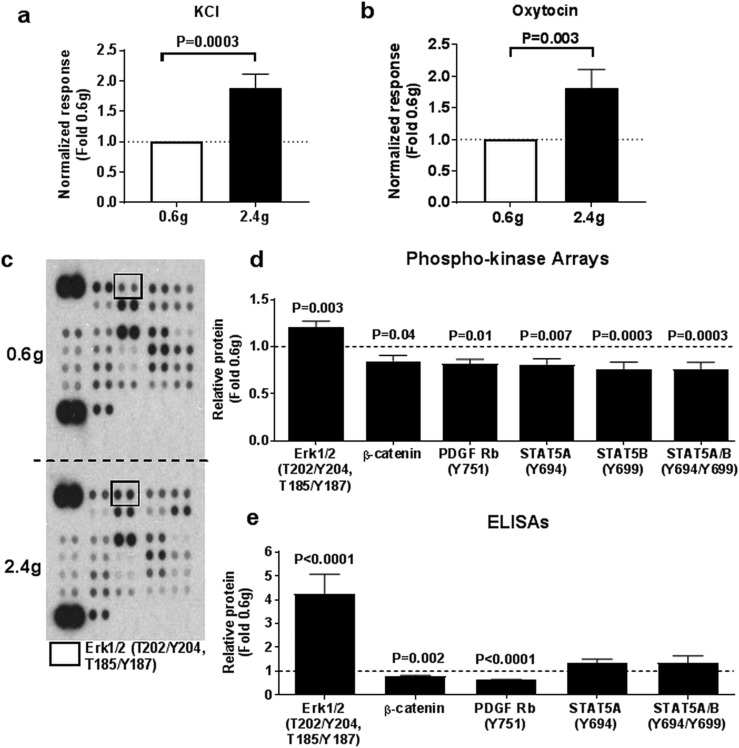
Effects of myometrial stretch on maximal contractile responses and activation of signaling proteins. Myometrial explants were incubated under low (0.6 g) or high (2.4 g) tension in duplicate and the maximal response to (a) KCl (50 mM) and (b) increasing concentrations of oxytocin (up to 100 nM) determined by isometric tension studies and expressed as fold change from 0.6 g tension. Values shown are mean + standard error, N = 10. Tissue lysates of explants incubated under low (0.6 g) or high (2.4 g) tension and analyzed by (c, d) phosphokinase arrays and (e) tissue ELISAs. (c) Representative blots showing the levels of phosphorylation of individual kinases and their protein substrates in 0.6 g or 2.4 g tension tissues. Black boxes on the arrays correspond to ERK1/2 (T202/Y204, T185/Y187) antibodies. (d) Protein levels of targets significantly altered by stretch on phosphokinase arrays. Values shown are mean + standard error, N = 12, and were corrected for multiple testing. (e) Phosphokinase array results were validated in an independent cohort by tissue ELISAs using the same monoclonal antibodies where available. Values shown are mean + standard error, N = 10. Effect of stretch (2.4 g) on protein levels is expressed as fold change from low tension (0.6 g).

We initially sought to replicate our previous findings (11, 24) that prolonged mechanical stretch of human myometrium stimulates its contractility. Human myometrial biopsies were obtained at the time of planned cesarean section and divided into multiple explants from the same woman. Explants were repeatedly washed, and then pairs of explants from the same woman were incubated under high or low tension for 20 hours and their contractility subsequently studied *in vitro*. Incubation under high tension increased maximal responses to KCl by 90% and to oxytocin by 80% (*P* = 0.0003 and *P* = 0.003, respectively, N = 10; Fig. 1a and 1b; Supplemental Fig. 1a). The pEC_50_ to oxytocin (the −log_10_ of the molar concentration of the drug required to achieve 50% of the maximum response) was not affected by myometrial stretch (Supplemental Fig. 1b).

To examine the phosphorylation events triggered by prolonged stretch, we used a phosphokinase profiler array to compare levels of phosphorylated proteins in paired explants (from 12 additional women) incubated under high or low tension for 20 hours (Fig. 1c). We identified significantly increased ERK1/2 (T202/Y204, T185/Y187) phosphorylation, decreased *β*-catenin levels, and decreased phosphorylation of PDGF Rb (Y751) STAT5A (Y694), STAT5B (Y699), and STAT5A/B (Y694/Y699) (*P*_adj_ < 0.05; Fig. 1d). To validate the phosphokinase array results, we performed ELISAs in biological replicates (Fig. 1e), *i.e.*, using myometrial biopsies from an additional 10 women, as well as technical replicates (Supplemental Fig. 2), *i.e.*, using the same myometrial explants used for arrays. Again, exposure of explants to high tension for 20 hours significantly altered ERK1/2 (*P* < 0.0001) and PDGF Rb (*P* < 0.0001) phosphorylation and *β*-catenin protein levels (*P* = 0.002). The biggest proportional change was in ERK1/2 phosphorylation, which increased fourfold under high tension. Stretch-induced phosphorylation of STAT5A (Y694) and STAT5A/B (Y694/Y699) was not replicated when measured by ELISAs, and we were unable to validate the results of STAT5B (Y699) due to the lack of a reliable commercially available antibody for ELISA. Similar findings were also found in technical replicates of myometrial tissues used for phosphokinase arrays (Supplemental Fig. 2).

### Pharmacological antagonism of MEK–ERK pathway inhibits stretch-induced myometrial contractile activity

**Figure 2.**
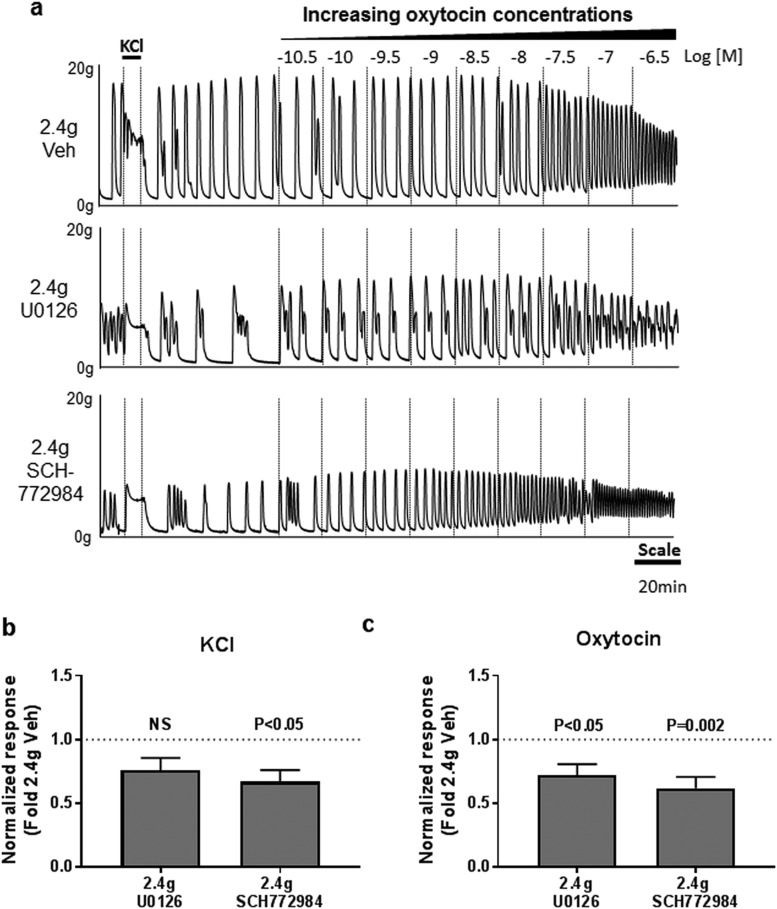
Effect of MEK1/2 and ERK1/2 inhibitors on the maximal contractile responses to KCl and oxytocin. Myometrial explants were incubated under high tension (2.4 g) with 10 µM U0126 (MEK1/2 inhibitor), 10 µM SCH772984 (ERK1/2 inhibitor), or vehicle (Veh) in duplicate. (a) Representative set of traces showing the response to KCl (50 mM) and increasing concentrations of oxytocin (up to 100 nM). The effect of 2.4 g vehicle, 2.4 g U0126, or 2.4 g SCH772984 on maximal contractile responses to (b) KCl or (c) oxytocin, expressed as fold change from 2.4 g vehicle. Values shown are mean + standard error, N = 10.

The functional significance of stretch-induced ERK activation was investigated by incubating explants from an additional 10 women under high or low tension for 20 hours with or without ERK inhibitors. We targeted ERK activity using two pharmacological antagonists of the MEK–ERK signaling pathway: U0126 was used to inhibit MEK1/2, the upstream kinase of ERK1/2, whereas SCH772984 is a competitive inhibitor of the ERK1/2 kinase. The effect of stretch on the KCl response was inhibited by SCH772984 by 36% (*P* < 0.05; Fig. 2a; Supplemental Fig. 3a), whereas both U0126 and SCH772984 prevented stretch-mediated increase in oxytocin response by 37% and 44%, respectively (*P* < 0.05 and *P* = 0.002, respectively; Fig. 2b; Supplemental Fig. 3a). The pEC_50_ values to oxytocin were not significantly affected by any of the treatments (Supplemental Fig. 3b).

### Concentration dependence of retosiban in regulating stretch-induced myometrial contractility

**Figure 3.**
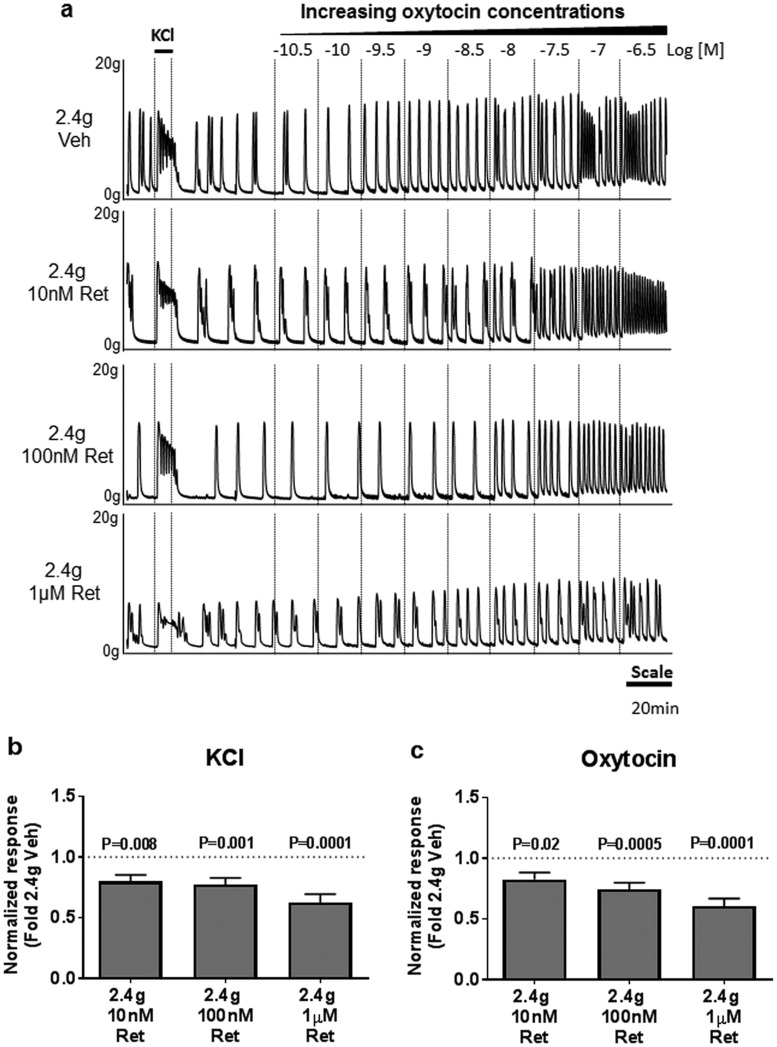
Effect of retosiban (Ret) on maximal contractile responses to KCl and oxytocin. Myometrial explants were incubated under high tension (2.4 g) in the presence of 10 nM, 100 nM, or 1 µM retosiban, or vehicle (dimethyl sulfoxide) in duplicate. (a) Representative set of traces showing the response to KCl (50 mM) and increasing concentrations of oxytocin (up to 100 nM). The effect of retosiban on maximal contractile responses to (b) KCl or (c) oxytocin, expressed as fold change from 2.4 g vehicle controls. Values shown are mean + standard error, N = 20.

We then sought to replicate our previous finding that 1 µM retosiban (11) reduces the effect of prolonged stretch and to determine whether this effect was seen with lower, therapeutically relevant concentrations of the drug. Explants from the same myometrial biopsy were incubated under high stretch with retosiban or its vehicle for 20 hours. All explants were then thoroughly washed to remove the drug, and their contractility was studied *in vitro*. Incubation in retosiban (10 nM, 100 nM, and 1 µM) during the period of mechanical stretch was associated with decreased subsequent maximal responses to KCl (21%, 23%, and 38%, *P* = 0.008, *P* = 0.001, and *P* = 0.0001, respectively; Fig. 3a; Supplemental Fig. 4a) and oxytocin (18%, 25%, and 40%, *P* = 0.02, *P* = 0.0005, and *P* = 0.0001, respectively; Fig. 3b; Supplemental Fig. 4a). The pEC_50_ of myometrial explants to oxytocin was not significantly affected by retosiban at these concentrations (Supplemental Fig. 4b).

### Effect of retosiban on stretch-induced phosphokinase proteins

**Figure 4.**
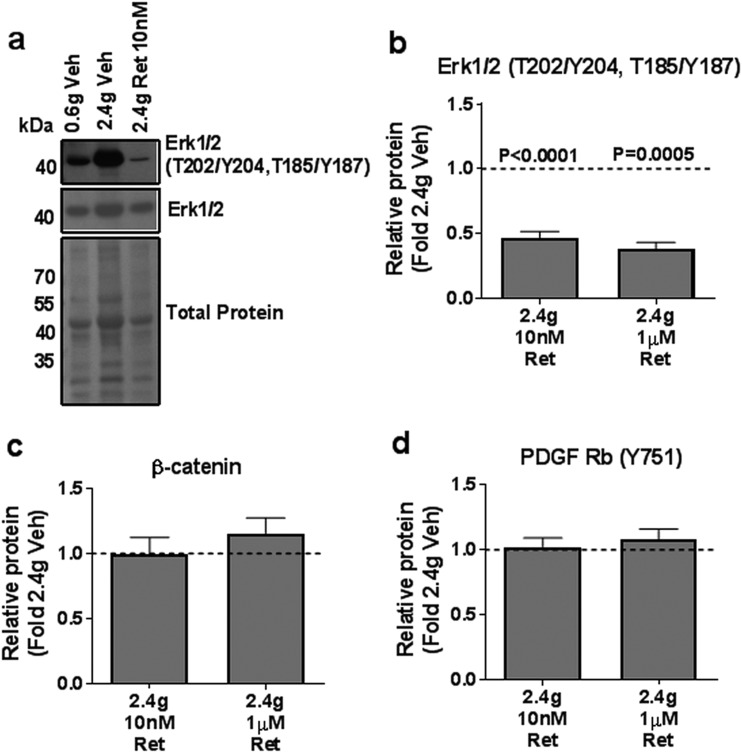
Retosiban effects on phosphorylated and total protein levels of ERK1/2, *β*-catenin, and PDGF Rb. Protein levels of tissue lysates of explants incubated under high tension (2.4 g) with vehicle, 10 nM or 1 µM retosiban. (a) Representative Western blot of phospho- and total ERK1/2 in tissues under low or high tension vehicle or high tension with 10 nM retosiban. Histograms represent relative protein levels of (b) phospho-ERK1/2 (T202/Y204, T185/Y187), (c) *β*-catenin, and (d) phospho–PDGF Rb (Y751). Data are represented as fold change from high tension vehicle (2.4 g Veh). Values shown are mean + standard error, N = 10.

To determine the mechanism of action of retosiban, we exposed explants from an additional 10 women to 20 hours of high tension in the presence of the drug or vehicle and then analyzed the signaling proteins that we had identified as regulated by stretch. Both 10 nM and 1 µM retosiban reduced phosphorylation of ERK1/2 (Fig. 4a and 4b), by 53% and 62%, respectively (*P* < 0.0001 and *P* = 0.0005). In contrast, retosiban had no effect on *β*-catenin protein and phospho–PDGF Rb levels (Fig. 4c and 4d). Lower concentrations of retosiban (0.1 nM and 1 nM) did not affect stretch-induced ERK1/2 phosphorylation (Supplemental Fig. 5).

### Comparison of the effects of retosiban and atosiban on stretch-induced ERK1/2 phosphorylation

**Figure 5.**
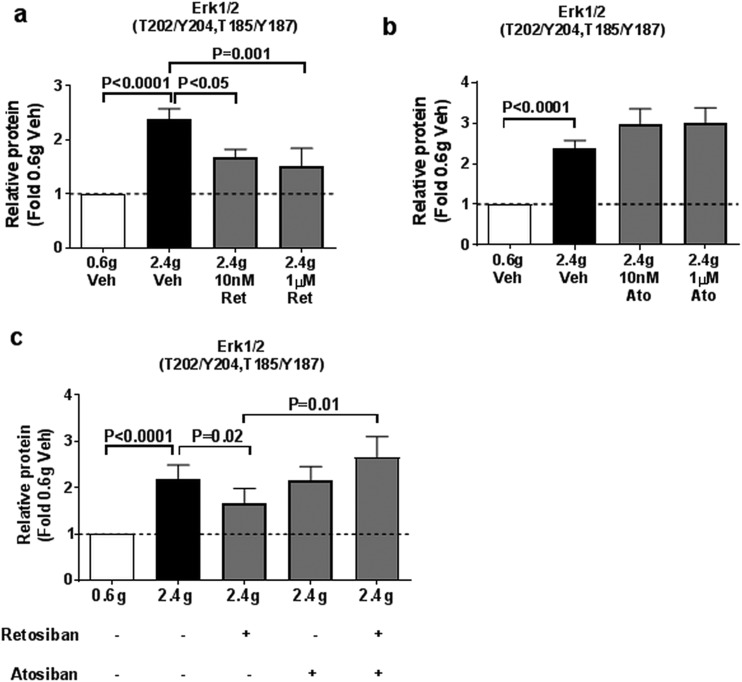
Effect of retosiban, atosiban (Ato), or retosiban with atosiban on ERK1/2 phosphorylation in stretched tissues. Myometrial explants were incubated under low tension with vehicle, high tension with vehicle, high tension with (a) retosiban (10 nM or 1 µM), or high tension with (b) atosiban (10 nM or 1 µM). Tissue lysates were examined for ERK1/2 phosphorylation by ELISAs. Data are represented as fold change from low tension vehicle. Values shown are mean + standard error, N = 6 to 13. (c) Myometrial explants incubated under high tension were treated with vehicle, 10 nM retosiban, 1 µM atosiban, or 10 nM retosiban and 1 µM atosiban. Tissue lysates were examined for phospho-ERK1/2 (T202/Y204, T185/Y187) levels by ELISAs. Data are represented as fold change from low tension vehicle. Values shown are mean + standard error, N = 20.

We next studied ERK1/2 phosphorylation in myometrial explants incubated under high stretch and compared the effects of retosiban with atosiban, a structurally unrelated peptide antagonist of the OTR (N = 6 to 13). Again, stretch-induced ERK1/2 phosphorylation was significantly inhibited by 10 nM retosiban (*P* < 0.05) and 1 µM (*P* = 0.001) retosiban (Fig. 5a). Interestingly, atosiban did not inhibit stretch-induced ERK1/2 phosphorylation at concentrations up to 1 µM (Fig. 5b).

### Atosiban reverses the inhibitory effects of retosiban on stretch-induced ERK1/2 phosphorylation

To establish whether the inhibitory effect of retosiban on stretch-induced myometrial contractility was mediated through the OTR, we determined whether this effect of retosiban could itself be blocked by a 100-fold molar excess of atosiban. We studied myometrial biopsies from an additional 20 women. We again observed that prolonged stretch of myometrial explants increased ERK1/2 phosphorylation, and that this effect was inhibited by coincubation in 10 nM retosiban. Atosiban (1 µM) alone did not significantly alter ERK1/2 phosphorylation in tissues incubated under low tension (with or without retosiban) or high tension in the absence of retosiban (Fig. 5c; Supplemental Fig. 6). However, in explants incubated under high tension, coincubation with atosiban prevented the inhibitory effects of retosiban on ERK1/2 phosphorylation (*P* = 0.01) (Fig. 5c).

### Mechanical stretch does not affect myometrial oxytocin levels

To determine whether the effects of stretch on oxytocin receptor activity were associated with changes in myometrial oxytocin production, we measured oxytocin levels in myometrial explants incubated under low and high tension (N = 16). Myometrial oxytocin levels were not affected by stretch (Supplemental Fig. 7).

### Retosiban reduces the risk of spontaneous labor in cynomolgus monkeys

**Figure 6.**
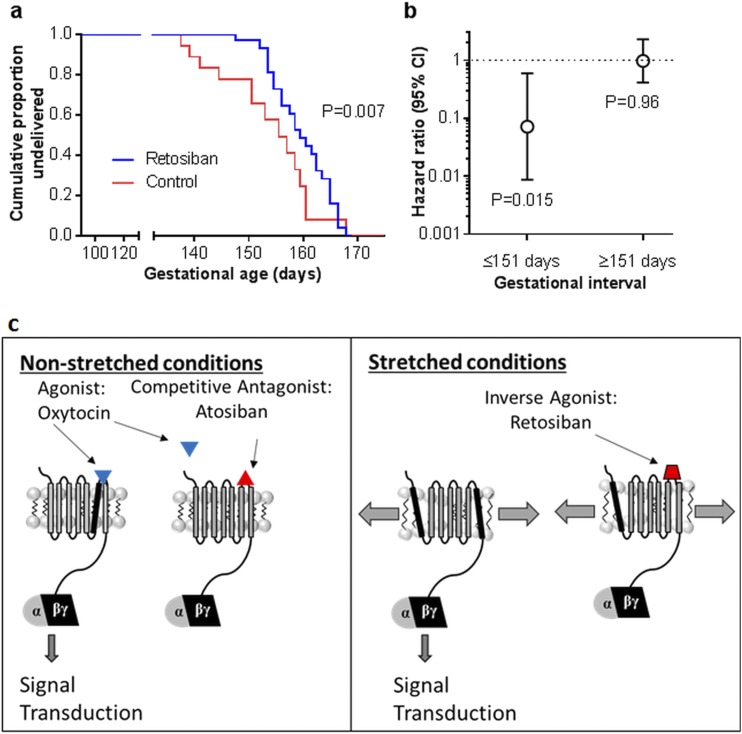
Effect of retosiban (100 mg or 300 mg) between 100 and 150 days of gestational age on the timing of spontaneous labor in the cynomolgus monkey, and model of OTR conformational states under nonstretched and stretched conditions. (a) Time-to-event analysis treated spontaneous delivery as the event and prelabor cesarean delivery as censoring, and the *P* value was estimated using the Wilcoxon (Breslow) test for equality of survivor functions, which does not assume proportional hazards. The result was also statistically significant when compared using the Tarone-Ware test and the Peto-Peto-Prentice tests (*P* = 0.02 and 0.01, respectively), which also do not assume proportional hazards. (b) The hazard ratio for delivery associated with retosiban treatment from 100 to 150 days, splitting the period of analysis at day 151. The test of the proportional hazards assumption indicated that the hazard of spontaneous delivery associated with being treated by retosiban varied over the entirety of the experiment (*P* = 0.02). When we repeated the analysis splitting the data on the day of cessation of treatment with retosiban (day 151), there was no evidence for nonproportionality of the hazard ratio when the two time periods were analyzed separately: 100 to 151, *P* = 0.60; 151 to 176, *P* = 0.53, but the risk of spontaneous labor only differed in relation to treatment group over the period (up to 150 days) when the animals actually had the drug administered. (c) Hypothetical model of OTR conformational states triggered by mechanical stretch. In nonstretched conditions, oxytocin changes the conformational state of the OTR leading to signal tranduction. This effect can be inhibited by preventing oxytocin binding to the OTR by antagonists or inverse agonists. Mechanical stretch promotes agonist-free conformational changes in the OTR, which activate signal transduction. This effect of stretch can be inhibited by inverse agonism of the OTR.

To establish whether oral retosiban could reduce the risk of spontaneous labor, we reanalyzed an extensive series of toxicological experiments performed in a pregnant nonhuman primate model, the cynomolgus monkey. The analytic approach was to use time-to-event analysis to compare the probability of spontaneous labor, as previously described in women (18). The event was spontaneous delivery, and prelabor cesarean was treated as censoring. Animals were exposed to vehicle (n = 18), 100 mg/kg oral retosiban (n = 18), or 300 mg/kg oral retosiban (n = 18), and the two doses of retosiban were combined into a single group. A Kaplan–Meier plot (Fig. 6a) indicated an excess of early deliveries in vehicle-treated animals, and there was a statistically significant difference between the two curves (*P* = 0.007). The association remained significant when the single animal delivered by emergency cesarean was excluded (*P* = 0.003). The two doses of retosiban had been combined into a single group to increase statistical power. However, when the two doses were analyzed individually, there were significant associations with both 100 mg/kg (*P* = 0.045) and 300 mg/kg (*P* = 0.026). When all animals delivered by cesarean section were excluded, there was still a significant association with retosiban (*P* = 0.01).

Analyzing all retosiban-treated animals, the test for nonproportionality of the hazards across the whole range of gestational age was significant (*P* = 0.02). In the earlier period (*i.e.*, during treatment), the retosiban group had a reduced risk of spontaneous (hazard ratio = 0.07, 95% confidence interval 0.01 to 0.60, *P* = 0.015), whereas there was no difference in the later period (*i.e.*, after cessation of the treatment; Fig. 6b). Collectively, the data demonstrate a reduced risk of early labor among animals while being treated with the drug, but no difference in the timing of normal labor after its cessation.

## Discussion

We confirm that prolonged stretch of human myometrium increases its contractility. This property of uterine smooth muscle is biologically and clinically relevant as it is thought to be involved in the physiological control of parturition at term and may be a determinant of the much higher rates of preterm birth observed in multiple pregnancies. In the current study, we provide mechanistic evidence for why stretch has this effect and identify a therapeutic approach to reverse it. Furthermore, we provide evidence that oral retosiban prevented early delivery in a nonhuman primate.

We show that prolonged stretch of human myometrium increases phosphorylation of a number of signaling proteins, with the strongest effect being increased phosphorylation of ERK1/2. The functional importance of ERK1/2 phosphorylation is illustrated by the prevention of stretch-induced contractility by coincubation with two established inhibitors of the ERK1/2 signaling pathway. In multiple biological replicates, we have demonstrated that the stimulatory effect of stretch on myometrial contractility is prevented by coincubation in retosiban, an OTR antagonist. Moreover, retosiban has this effect in low nanomolar concentrations of the drug, which are well within the range obtained therapeutically (25), and the inhibition of stretch-induced contraction is paralleled by the inhibition of stretch-induced phosphorylation of ERK1/2. Finally, the effect of retosiban on ERK1/2 phosphorylation is itself blocked by a 100-fold molar excess of atosiban, a structurally unrelated competitive antagonist of the OTR. Collectively, the data support the hypothesis that myometrial stretch causes agonist-free activation of the OTR and that retosiban reverses this effect through inverse agonism of the same receptor. Furthermore, it is unlikely that the effects of retosiban were due to antagonism of endogenous oxytocin as (1) atosiban had no such effect and (2) there were no differences in oxytocin levels in tissues incubated under low or high tension.

Three lines of evidence indicate that the effects of stretch and retosiban are mediated through the OTR. First, the effects of retosiban were observed in the nanomolar range, making it unlikely that these were off-target effects of the drug. Second, ERK1/2 is known to be a key signaling pathway following OTR activation (26). Third, the effects of retosiban were blocked by an excess of a structurally unrelated OTR antagonist. Furthermore, the plausibility of the hypothesis is supported by other previous studies. Multiple other studies of myometrium have demonstrated effects of stretch on the OTR and ERK1/2 (10, 27, 28). Moreover, stretch-induced receptor activation and reversal by inverse agonists have previously been described in other tissues and with other G protein–coupled receptors. For example, analogous observations have been made in cultured rat myocardiocytes, where mechanical stretch increased ERK1/2 phosphorylation by agonist-free activation of the AT1 receptor, which was reversed by an orally active nonpeptide AT1 receptor antagonist (olmesartan) through an additional inverse agonist property of the drug (29–31). There are many other examples of mechanosensitive G protein–coupled receptors (32), in particular a number of G_q/11_-coupled receptors appear to function as mechanosensors in smooth muscle cells (33). Hence, the data support the concept that the OTR, which is also a G_q/11_-coupled receptor, acts as a uterine mechanosensor in an oxytocin-independent manner (illustrated in Fig. 6c). We speculate that this property of the oxytocin receptor is involved in the physiological initiation of parturition at term.

On the basis of these findings, we speculate that retosiban could have utility as a preventative treatment in multiple pregnancy. The increased risk of spontaneous preterm birth in twins is thought to be related to stretch-induced stimulation of myometrial contractility. The current data indicate that retosiban may specifically inhibit this effect. If so, it is plausible that retosiban could be given as a preventative treatment, as stretch is also thought to be involved in the control of parturition near term. Hence, it is plausible that the drug could also reduce the risk of labor if administered in the period of gestation leading up to term. This is not a hypothesis that can be tested in women. However, we reanalyzed an extensive series of toxicological experiments performed in a nonhuman primate model (these were a requirement from the Food and Drug Administration prior to clinical studies in pregnant women). A complexity in the approach to that dataset was the fact that 15 of 54 animals were delivered by prelabor, planned cesarean as part of the experimental design. We addressed this using time-to-event analytic methods that account for censoring. This allows the animal to be included in the denominator in the weeks leading up to the day of planned cesarean. Simply dropping these animals both reduces statistical power and systematically excludes animals that did not experience early labor. Using these methods, we found strong evidence to support the hypothesis that oral retosiban reduced the risk of early labor. However, these results should be assessed cautiously as this was a secondary analysis of data, *i.e.*, determining the effect of retosiban on the timing of labor was not the primary purpose of the primate experiments. Moreover, we analyzed the effect of retosiban on the timing of labor in the last third of pregnancy. The rationale is that increased myometrial stretch might be involved in the association between spontaneous preterm birth in multiples and the physiological regulation of the timing of labor at term. Although the primate work supported our *in vitro* data, we do not have direct evidence from animal studies for a protective effect of the drug in the context of preterm labor and multiple pregnancy.

A Phase 2 clinical trial of intravenous retosiban for the treatment of spontaneous preterm birth in singleton pregnancies showed favorable efficacy and safety profile (25). Unfortunately, Phase 3 trials of the drug for the treatment of spontaneous preterm labor have recently been discontinued due to lack of recruitment. This may reflect the existence of multiple other agents for acute tocolysis and a generally poor evidence base supporting better infant outcomes in this context. However, high-risk multiple pregnancy (*e.g.*, twins with a short cervix or triplets and higher multiples) is a major area of unmet clinical need (7). Hence, a possible future approach to clinical evaluation of this drug would be to treat women with high-risk multiple pregnancy, potentially focusing on the gestational ages (24 to 32 weeks) associated with the highest risks of severe short- and long-term adverse outcome (34). Such a preventative approach is facilitated by the fact that the drug can be administered orally. Finally, unlike many other drugs that control uterine contractility, such as *β*2 adrenoceptor agonists or calcium channel blockers, retosiban does not have widespread maternal systemic effects, due to its high degree of specificity for the OTR and the limited role for this receptor in regulating other physiological processes.

## Supplementary Material

Supplemental Material

## Abbreviations:

ECL: enhanced chemiluminescence
ELISA: enzyme-linked immunosorbent assay
ERK: extracellular signal-regulated kinase
MEK: mitogen-activated protein kinase kinase
OTR: oxytocin receptor
PDGF Rb: platelet-derived growth factor receptor *β*
pEC_50_: negative logarithm of the half-maximal effective concentration
TBS-T: Tris-buffered saline containing 0.1% Tween.
