# Supplementary material for: Retosiban Prevents Stretch-Induced Human Myometrial Contractility and Delays Labor in Cynomolgus Monkeys
Source: J Clin Endocrinol Metab. 2017 Dec 26;103(3):1056–67. doi: 10.1210/jc.2017-02195 (PMC5868409; doi:10.1210/jc.2017-02195)
Supplement: Supplemental Material [file jc.2017-02195_supplemental_material.docx]

**SUPPLEMENTARY FIGURE LEGENDS**

**FIGURE S1. Effect of stretch on myometrial contractility.** Myometrial explants were incubated under low (0.6g) or high (2.4g) tension with vehicle (DMSO) in duplicate for 20h and isometric tension studies performed. (a) Representative set of traces showing the response to KCl (50mM) and increasing concentrations of oxytocin (up to 100nM). (b) pEC50 values were calculated using analysis of the area under the curve for each concentration to oxytocin, as described in the Methods. Values shown are mean+SE, N=10.

**FIGURE S2. Validation of phospho-kinase array results in technical replicates.** Myometrial explants were incubated under low (0.6g) or high (2.4g) tension for 20h and processed for lysates prior to phospho-kinase array analysis or tissue ELISAs. The same tissue lysates were used for both assays. Histograms represent relative protein levels of (a) Phospho-Erk1/2 (T202/Y204, T185/Y187), (b) β-catenin, (c) Phospho-PDGF-Rb (Y751) and (d) Phospho-STAT5A/B (Y694/699). Values shown are mean+SE, N=10 for all except (d) STAT5A/B, where N=4.

**FIGURE S3. Effect of MEK1/2 and Erk1/2 inhibitors on pEC50 to oxytocin in stretched myometrial tissues.** Myometrial explants were incubated under high tension (2.4g) with MEK1/2 inhibitor (U0126, 10μM), Erk1/2 inhibitor (SCH772984, 10μM) or vehicle (DMSO) in duplicate for 20h. pEC50 values were calculated using analysis of the area under the curve for each concentration to oxytocin, as described in the Methods. Values shown are mean+SE, N=10.

**FIGURE S4. Effect of retosiban on pEC50 to oxytocin in in stretched myometrial tissues.** Myometrial explants were incubated under high tension (2.4g) in the presence of 10nM, 100nM or 1μM retosiban, or vehicle (DMSO) in duplicate for 20h. pEC50 values were calculated using analysis of the area under the curve for each concentration to oxytocin, as described in the Methods. Values shown are mean+SE, N=10.

**FIGURE S5. Effect of low retosiban concentrations on Erk1/2 phosphorylation in high tension tissues.** Myometrial explants incubated under high tension were treated with vehicle, 1nM retosiban, 0.1nM retosiban or vehicle for 20h. Data are represented as fold change from high tension vehicle. Values shown are mean+SE, N=13.

**FIGURE S6. Atosiban and retosiban does not affect Erk1/2 phosphorylation in low tension tissues.** Myometrial explants incubated under low tension were treated with vehicle, 10nM retosiban, 1μM atosiban or 10nM retosiban and 1μM atosiban. Tissue lysates were examined for Phospho-Erk1/2 (T202/Y204, T185/Y187) levels by ELISAs. Data are represented as fold change from low tension vehicle. Values shown are mean+SE, N=20.

**FIGURE 7. Effect of stretch on myometrial oxytocin levels.** Myometrial explants incubated under low or high tension for 20h. Tissue lysates were processed for oxytocin ELISAs as described in Methods. Data are represented as fold change from low tension vehicle. Values shown are mean+SE, N=16.

**TABLE S1. Table of key resources.**

**
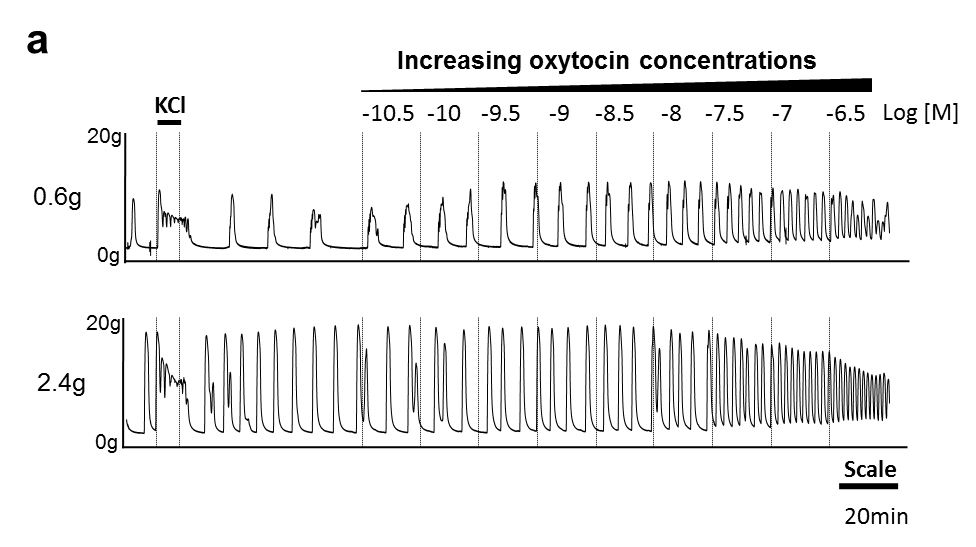
**

**
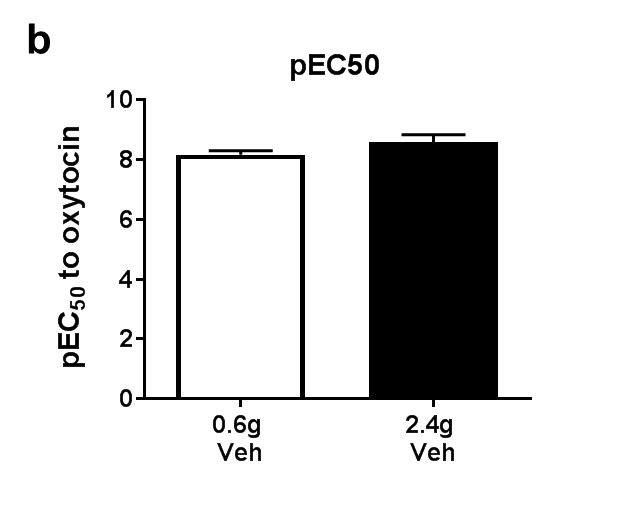
**

**FIGURE S1.**

**FIGURE S2.**

**FIGURE S3.**

**FIGURE S4.**

**FIGURE S5.**

**FIGURE S6.**

**FIGURE S7.**

| **REAGENT** | **SOURCE** | **IDENTIFIER** |
| --- | --- | --- |
| Proteome Profiler Human Phospho-Kinase Array Kit | R&D Systems | ARY003B |
| Phospho-ERK1 (T202/Y204)/ERK2 (T185/Y187) DuoSet IC ELISA | R&D Systems | DYC1018B |
| Human Total beta-Catenin DuoSet IC ELISA | R&D Systems | DYC1329 |
| Human Phospho-PDGF R beta (Y751) DuoSet IC ELISA | R&D Systems | DYC3096 |
| STAT5 alpha (Phospho) [pY694] Human ELISA Kit | Thermo Fisher Scientific | KHO0761 |
| STAT5 A/B (Phospho) [Y694/699] ELISA Kit | Abcam | AB176656 |
| Human/Mouse/Rat Phospho-ERK1 (T202/Y204)/ERK2 (T185/Y187) Antibody | R&D Systems | MAB1018 |
| Oxytocin ELISA kit | ENZO | ADI-900-153 |
| Anti-rabbit IgG, HRP-linked Antibody | Cell Signaling Technology | #7074 |

**TABLE S1.**
